# Neonicotinoid insecticide residues in subsurface drainage and open ditch water around maize fields in southwestern Ontario

**DOI:** 10.1371/journal.pone.0214787

**Published:** 2019-04-04

**Authors:** Arthur W. Schaafsma, Victor Limay-Rios, Tracey S. Baute, Jocelyn L. Smith

**Affiliations:** 1 Department of Plant Agriculture, University of Guelph—Ridgetown Campus, Ridgetown, Ontario, Canada; 2 Ontario Ministry of Agriculture Food and Rural Affairs, Ridgetown, Ontario, Canada; University of Pittsburgh, UNITED STATES

## Abstract

Neonicotinoids are widely used class of insecticides. Most are seed treatments and during planting active ingredient may be abraded and lost in fugitive dust. Much of this active ingredient contaminates surface waters, exposing aquatic organism to potential ill effects. This study examines concentrations of neonicotinoids appearing in tile drains and open ditches around commercial maize fields around planting time where neonicotinoid seed treatments had been used. This sample set represents surface water leaving the point of origin, for which data are sparse. Clothianidin was found more often than thiamethoxam and at higher concentrations; at a median concentration of 0.35 ng/mL in tile drain water and almost twice that (0.68 ng/mL) in ditches into which the tiles are draining after applications of 19 g/ha on seed. This concentration reveals a 40 to 50 fold dilution for neonicotinoid residues between the points where they leave the field in which they were applied and when they are found in nearby streams in a similar ecosystem. Our data support that for a no-observed-effect concentration of 0.3 ng/mL for thiamethoxam there would be between a 1.6 and 100-fold margin of safety to mayflies in most streams if fugitive dust on pneumatic planters were properly mitigated.

## Introduction

Neonicotinoids globally are used as insecticidal seed treatments [1–3]. Clothianidin and thiamethoxam are the neonicotinoids most used today in southwestern Ontario and these are used as seed treatments commonly on maize, soybean, and canola [4]. Only a small fraction of the neonicotinoid active ingredient is absorbed by the target crop when applied as a seed treatment [5, 6], with the remainder entering the soil or degrading [7].

Neonicotinoid residues in soils decline rapidly after application by processes of plant absorption, degradation, sorption to soil particles and leaching [3, 8]. Some argue that their solubility in water makes them strong candidates for leaching resulting in surface water contamination [9–11]. Others argue that despite their solubility, other chemical properties such as time dependent sorption make them relatively immobile once in soil [12].

The coincidence between honey bee mortality, the widespread adoption of neonicotinoid insecticides as seed coatings, and the potential unintended effects on other non-target organisms has attracted significant attention [13–15]. More recently, neonicotinoid residues have been found in surface water surrounding agricultural fields [16–22]. Some suggest neonicotinoid residues in surface water may affect aquatic invertebrates and ecosystem health [17, 23–26]. Of these invertebrates, mayflies (Ephemeroptera)[27, 28] and midges (Diptera: Chironomidae) [29] are among the most sensitive species and could be used as sensitivity benchmarks to neonicotinoid-contaminated surface waters. To understand exposure of these sensitive species, it is important to determine the pathways by which neonicotinoid residues reach the surface waters in question.

As already noted, many believe that the mobility and leaching potential for neonicotinoid insecticides in soil solution is the major cause of contamination of surface waters. Agricultural chemical leaching varies greatly by soil texture, organic matter content, precipitation, formulation, and insecticide application method [30]. Imidacloprid was observed to leach down to a 60-cm depth under field conditions of low organic matter, sandy soil texture, alkaline pH, and low cation exchange capacity [31]. Commercial formulations of imidacloprid tend to have a higher leaching potential than analytical grade ingredient [32] which may be due to the presence of adjuvants. Clothianidin is mobile to highly mobile in the laboratory, but only a minor amount of leaching was observed in field studies [33]. In two field studies in Germany, only the degradants of clothianidin were detected in small quantities in the leachate, and parent material was not detected [33]. When thiamethoxam was applied to 30-cm columns of four soil textures (loamy sand, sand, loam, silt loam) with water equivalent to 200 mm of precipitation, the recovery rate of thiamethoxam in the leachate for these soil textures were 1.6, 23, 0.6% and below the level of detection, respectively [34]. However, under simulated heavy rainfall conditions (equivalent to 65 cm rainfall), 66–79% of the applied thiamethoxam was recovered from leachate in a 25-cm sandy loam soil column [35]. More recently in a laboratory soil column study, thiamethoxam was detected in leachate after a major rainfall event was simulated at the end of the study, while none was detected in regular smaller rainfall events during the course of the experiment [11]. The published data suggest that leaching is a likely phenomenon in field situations; however, it is unclear whether leaching is a major contributor to surface water contamination.

Macropores, such as surface cracks, earthworm burrows or root channels, can also connect tile drains to the surface, increasing the potential for tile water contamination through preferential flow [36, 37]. In southwestern Ontario, most agricultural fields have subsurface tile drainage systems installed to increase the opportunity to produce sustained high yields with a variety of crops. Subsurface drainage is typically placed at a depth of 60–120 cm with 20–50 m spacing [38].The subsurface tile drainage system may increase the development of soil macropores systems which increase the possibility of applied nutrients and pesticides to reach tile drains by preferential flow [38].

If leaching is the main source of contamination of flowing surface water from neonicotinoid seed-treatment application, it would follow that in general, neonicotinoid concentrations in drainage tile effluent would be higher than in ditches into which they drain. In the first report of neonicotinoid concentrations at field edges in moving water coming from tile drainage, Chrétien et al [21] showed that thiamethoxam concentrations were four times higher in surface run-off water than in tile effluent, suggesting that the majority of thiamethoxam was coming from the soil surface. They did not measure thiamethoxam concentrations in the ditches into which the tiles emptied.

In a study of commercial fields planted with neonicotinoid-treated corn seed in southwestern Ontario in 2013, we measured neonicotinoid concentrations in water samples flowing out of tile drains and in water collected from ditches into which the tiles were draining. One third of these data were reported earlier [19] as part of a larger data set focussed on standing water (puddles) within and around seed-treated fields as sources of exposure to foraging honey bees. We had collected a large number of additional running ditch and tile water samples at the same time which have subsequently been analyzed; and these data have not been reported elsewhere. We needed to publish these new data in response to the growing interest in sources of exposure of aquatic invertebrates to neonicotinoid residues in surface water originating from agricultural fields[9]. These new data compliment those recently reported by Struger et al. [39] for streams in the same region as our study and during a similar time period, allowing a calculation of a dilution factor between source and stream not possible before.

We hypothesized that neonicotinoid concentrations in ditches would be higher than those found in tile effluent, suggesting that sources of neonicotinoid contamination other than leaching from treated seed contributed to surface water contamination. Pneumatic planters, adopted widely in North America for their seed placement precision and efficiency, have been identified as a source of dispersion of abraded seed coating during planting [40] that can contaminate air, vegetation, surface soil and water in surrounding fields, which we believe is an important source of surface water contamination. Our objective was to investigate the quantity, distribution and temporal dynamics of clothianidin and thiamethoxam in tile and ditch water connected with drainage systems related to corn production fields. We propose that mitigating fugitive dust during planting of corn seed treated with pesticides, including neonicotinoids, would make a significant contribution to reducing contamination of surface water from seed treatment pesticides.

## Materials and methods

### Study fields and water sample collection

As part of a larger study [19] on honey bee exposure to residues related to neonicotinoid-treated corn seeds in corn fields, nine locations across Essex, Chatham-Kent, Lambton, Middlesex and Elgin Counties in southwestern Ontario were selected for study in 2013. Each location contributed two commercial corn fields. Both fields at each location were planted using the same seed source, the same planting equipment and the same operator. The only difference between the two fields was that one had seed lubricated by adding talc, which is known to be abrasive and leads to more fugitive contaminated dust [15], and the other had seed lubricated using a Bayer Fluency Agent [41] (Bayer CropScience, Calgary, AB). Water collection methods were reported in Schaafsma et al.[19]. Briefly, study fields were surveyed weekly from 29 April to 28 June 2013 for potential water sampling sites. Water samples were categorized as puddles of standing water within the perimeter of the field and “outside” of the study fields (results reported in Schaafsma et al. [19], and ditches or field drainage outlets (the subject of this paper). Fields and their surroundings were visited at weekly intervals and we sampled opportunistically each time ie, if there was water in a ditch we sampled it and if tiles were running we did as well. Not all instances had ditches and tiles in the same field, nor were all tiles always running at the time of sampling. Approximately 100 ml of water was collected for each sample in a new 100-ml amber HDPE bottle. Samples were placed immediately into a dark picnic cooler containing freezer packs for transport back to the laboratory, followed by immediate dark and frozen storage (-20°C) until analysis.

### Chemical analysis

Sample extraction and neonicotinoid residues were determined using the liquid chromatography coupled with tandem mass spectrometry (LC-MS/MS) method previously reported [19], except extracts were dried down with a Rapidvap Vertex Dry Evaporator (Labconco Corporation, Kansas City, MO) set at 40°C with a gentle nitrogen stream. Levels of detection and quantification (LOD and LOQ, respectively) were calculated as the mean peak height that could be detected using the mean height of the noise signal plus 3 and 10 × the standard deviation, respectively, around the analyte retention time and results are reported in S1 Table. Recovery tests were performed in triplicate by spiking a homogenized sample of 5 ml of blank water samples with the appropriate volume of analytical standards at 0.5 ng/ml. Spiked samples were allowed to equilibrate for 3 d at 40°C in darkness followed by our standard extraction procedure and analysis [19]. Determinations were made by injecting a 50-μL aliquot of extract into a 150-mm Gemini C18 reverse phase column (Phenomenex, Torrance, CA) with an Agilent 1100 Series high performance liquid chromatography (HPLC) (Agilent Technologies, Santa Clara, CA, USA). Eluates from the HPLC were introduced to an Ionics EP 10+ modified API 365 triple quadruple mass spectrometer (AB SCIEX, Concord, ON) system equipped with an electrospray ionization source (ESI). The source gas temperature was set to 550°C, nitrogen curtain gas 80 psi, nebulizer gas 8, collision gas 2, and ionization voltage 5000 V. Each compound was analysed in positive ion polarity mode using a multiple reaction monitoring procedure. MS/MS was operated in multiple-reaction-monitoring (MRM) mode in positive polarity. All analytes were monitored for the precursor ion and one qualifier and one quantifier transition. The transition with the greater peak height and area was used for quantification. S1 Table summarizes the optimized MS/MS parameters for each compound included in this study. Clothianidin, thiamethoxam, imidacloprid, and acetamiprid were determined using their respective deuterium-labeled internal standard to wit clothianidin-d3, thiamethoxan-d3, imidacloprid-d4, acetamiprid-d3, respectively (Sigma-Aldrich, St. Louis, MO, USA; Pestanal class, purity ≥99.5%). Because no commercially available internal standards were available for thiacloprid, dinotefuran and nitenpyram, the external calibration procedure was used by preparing matrix-matched calibration curves at 9 concentrations from 0.06 to 4.00 ng/mL and injected directly in the LC-ESI (+)-MS/MS system.

### Statistical methods

Simple descriptive statistics such as raw means, medians, maximums, standard error, and percentage of samples with compound detected were calculated for all compounds tested in Microsoft Excel. Generalized linear mixed model analysis was conducted for the fixed effects of water source, week of sampling, and, seed lubricant, and their interactions on the concentration of clothianidin in water using PROC GLIMMIX in SAS 9.4 (SAS Institute Inc., Cary, NC) [42]. Field location was considered a random effect. s. PROC UNIVARIATE and the Shapiro–Wilk statistic were used to test residuals for normal distribution, and studentized residuals were calculated to test for outliers using Lund’s test [43]. As a consequence, data were normalized using the lognormal distribution and identity link function. Only one outlier was removed from the sampling data. Least squares means were estimated using the inverse link option and pairwise comparisons were made using Tukey–Kramer tests to limit experiment-wise error rates (α = 0.05) [43].

## Results and discussion

Recovery values ranged from 43.1 ± 3.0% for thiacloprid to 96.3 ± 7.2% for acetamiprid, with an average of 77.7 ± 4% (S1 Table). All values below LOD, before being corrected for recovery rate, were considered negative detections.

**Fig 1:**
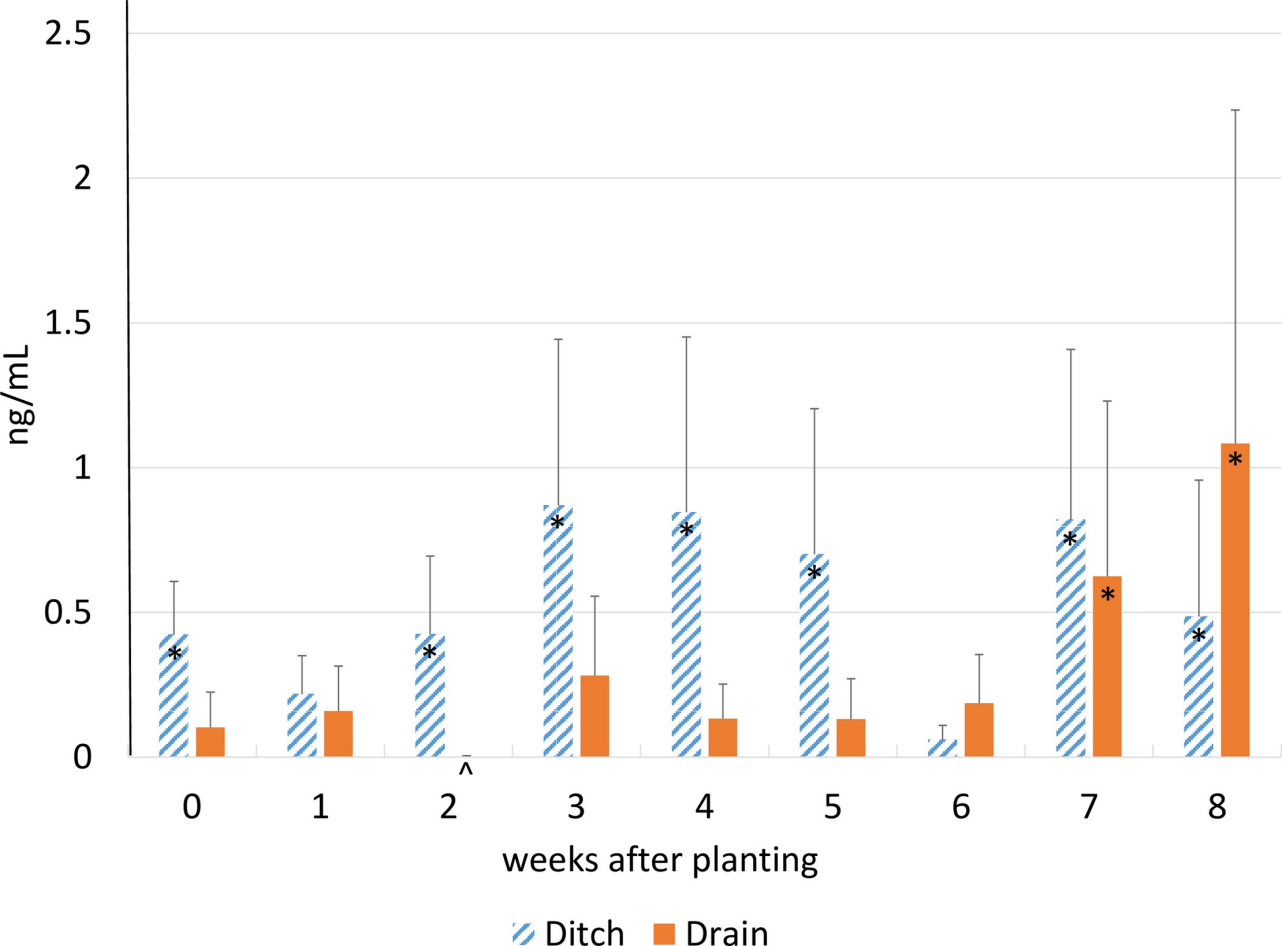
Mean concentrations of total (clothianidin plus thiamethoxam) neonicotinoid residues in ditch and drain water from maize (corn) fields that had been planted with neonicotinoid-treated seeds, southwestern Ontario, 2013, Bars are SE mean. Means with asterisk are significantly (P = 0.05) different by Tukey-Kramer test from the lowest (indicated with ^) mean value.

Clothianidin was the most common neonicotinoid detected in water leaving the study fields (Table 1). It was found in 88% of tile drain water samples and 95% of ditch samples (Table 1). Clothianidin was used as a seed treatment in more of the study fields than thiamethoxam. Furthermore, thiamethoxam breaks down into clothianidin [44], so it was not surprising that thiamethoxam was found less frequently. Imidacloprid was the third most commonly detected neonicotinoid insecticide, even though it occurred at concentrations near the LOD. The average concentration of several other neonicotinoid insecticides including imidacloprid, thiacloprid, acetamiprid, dinotefuran, and nitenpyram were at or below 0.02 ng mL^-1^ (Fig 1), which were considered as relatively minor and not subjected to further analysis in the present study. Imidacloprid was not used on any of the subject fields but may have been used in the region surrounding the study fields as a foliar spray for several crops, for flea control or for home and garden use [45]. Thiacloprid is registered for use on pome fruits as a foliar spray [45] and there were apple orchards near two of the study fields. Acetamiprid is used as a foliar spray on fruit and vegetable crops [45] and three sites were in close proximity to fruit and field vegetable production where acetamiprid may have been used. Dinotefuran is not registered for use in Canada [45], so it’s detection in 2 and 3% of tile drain and ditch samples was unexpected. Nitenpyram was not detected in this study, and is not an agricultural pesticide [45] but is widely used as a veterinary medicine for flea control in dogs and cats. That several neonicotinoid insecticides other than those applied to the study fields were detected in surface water indicates that these compounds are quite mobile and can move from nearby fields. We postulate that this movement is most likely from drift after pesticide application, with residues landing on soil surfaces reaching tile water mainly by preferential flow [46] of run-off or drift residues landing in ditches.

**Table 1:** Concentration of neonicotinoid insecticides in water leaving tile drains and in drainage ditches on the edges of 18 commercial corn fields where neonicotinoid insecticides had been applied as a seed treatment, and the field had a history of neonicotinoid seed treatment use on either wheat or soybeans in previous years. Water samples were collected in southwestern Ontario, Canada, 2013.

Neonicotinoid (ng/mL)	Tile drains (n = 50)					Ditches (n = 119)				
	(%)^1^	Mean	SE^2^	Median	Max^3^	(%)^1^	Mean	SE^2^	Median	Max^3^
clothianidin	(88)	0.88	0.18	0.35	6.95	(95)	1.11	0.31	0.68	7.2
thiamethoxam	(58)	0.24	0.07	0.04	2.63	(50)	0.23	0.05	0.00	3.82
imidacloprid	(12)	0.01	0.00	0.00	0.21	(13)	0.03	0.02	0.00	2.94
thiacloprid	(2)	0.00	0.00	0.00	0.04	(2)	0.00	0.00	0.00	0.08
acetamiprid	(8)	0.03	0.03	0.01	1.53	(5)	0.00	0.00	0.00	0.03
dinotefuran	(2)	0.00	0.00	0.00	0.04	(3)	0.00	0.00	0.00	0.03
nitenpyram	(0)	0.00	0.00	0.00	0.02	(0)	0.00	0.00	0.00	0.03

^1^percentage of positive samples (>LOD)

^2^standard error of untransformed data

^3^maximun value

Chretien et al [21] showed a median concentration of 0.44 ng/mL thiamethoxam in tile water after an application rate of 116 g/ha on seed. Our results for clothianidin (Table 1) are slightly higher with a median concentration of 0.35 ng/mL in tile drain water and almost twice that (0.68 ng/mL) in ditches into which the tiles are draining with application rates of around 19 g/ha. Struger et al [39] reported on concentrations of neonicotinoids found in four watersheds (Bear Creek, Sydenham River, Nissouri Creek, Thames River) representing regions where our study was conducted within approximately the same time period (2012 to 2014). Across this region, the maximum concentration of clothianidin found in streams was 0.182 ng/mL and the mean concentration across these locations was 0.0225 ng/mL [39]. Comparing these values with the concentration of clothianidin observed in ditch water draining our study fields (maximum 7.2 ng/mL and mean 1.11 ng/mL) (Table 1), we calculated a dilution factor of approximately 40- and 50-fold for maximum and mean concentrations, respectfully. To our knowledge, this is the first time a dilution factor between source surface (ditch) and stream water has been reported for neonicotinoid insecticides applied as seed treatments in field crops, and will be helpful to predict the impact of efforts to mitigate fugitive planter dust on exposure to aquatic organisms in streams.

For clothianidin, we detected an interaction (*p* = 0.0462) between the source of water sample (ditch vs. tile drain) and the week of sampling (days after planting) (Table 2). For the first 5 weeks after planting, concentrations tended to be higher in ditches than in tile drains (Fig 1), whereas in weeks 7 and 8, concentrations in tile drains began to rise and were similar to those in ditches. Rainfall amounts in the study region were generally at or below normal in May (Table 3) which corresponds to planting time and higher than normal in June and July, which corresponds to 4 to 8 wk after planting. Higher concentrations of clothianidin were observed in ditches rather than drains in the period between planting and 5 weeks after planting (Fig 1), suggesting that contaminated fugitive dust and surface run-off contributed more to ditch water contamination than tile effluent. These data suggest that in a year when the spring is dry tile effluent may be more important later in the season with high levels of rainfall (Fig 1). In soil column studies [11], thiamethoxam leaching was minimal for the first 30 d of the experiment which included applications of 0.9 cm of water every 3 d in columns that started at field capacity. It then took 9 cm of simulated precipitation at the end of the study for 75 ng/mL of thiamethoxam to leach out of the 20-cm column of sandy soil.

**Table 2:** Generalized linear mixed model analysis of main and interactive fixed effects of water source (tile drain or drainage ditch), sampling week (weeks after planting), and seed lubricant (talc or Bayer Fluency Agent) with field location as a random effect, on clothianidin concentration in water samples taken from the edge of agricultural fields planted with neonicotinoid-treated corn seed (clothiandin or thiamethoxam) in southwestern, Ontario, Canada, 2013.

Fixed effect*	Num DF	Den DF	F Value	*Pr*>F
Water source	1	132	4.12	0.0444
Seed lubricant	1	132	2.4	0.1237
Water source× seed lubricant	1	132	3.51	0.0631
Sampling week	8	132	2.38	0.0197
Water source × sampling week	8	132	2.04	0.0462

*type III test of reduced model

**Table 3:** Total monthly precipitation (mm) with 30-year normal in parentheses at Environment Canada automated weather stations located near study fields from March to July 2013, southwestern, ON.

Precipitation	Harrow	Sarnia	Ridgetown	St. Thomas	London
March	12.6 (70.0)	11.5 (57.5)	70.3 (59.9)	33.2 (65.7)	39.9 (71.5)
April	106.2 (83.0)	160.4 (71.5)	59.5 (79.7)	118.7 (83.4)	138.2 (83.4)
May	56.7 (89.3)	86.9 (79.7)	82.6 (79.7)	27.2 (87.3)	105.3 (89.8)
June	161.6 (86.1)	131.0 (83.1)	51.5 (77.9)	76.3 (92.4)	117.2 (91.7)
July	213.5 (89.2)	159.9 (78.5)	92.6 (85.4)	86.7 (83.0)	88.7 (82.7)
**Coordinates**					
North	42°02’00	42°59’58	42°27’00	42°46’06	43°02’00
West	82°54’00	82°18’32	81°53’00	81°12’18	81°09’00

We suggest there are three sources of surface soil contamination of agricultural fields by neonicotinoid insecticides in order of importance: 1) fugitive dust residues settling after planting [47], 2) fugitive dust coming from other fields [48], and 3) residues from previous applications or seed reaching the soil surface through upward capillary flow [49]. Neonicotinoid concentrations in ditches were generally higher than those in tile drains, especially early in the sampling period (Fig 1), suggesting that soil surface residues reached the ditches primarily through wind and/or water erosion, and less by leaching through drains. We [47, 50] showed that talc and soil dust abraded up to 12.6% of the active ingredient from seed during pneumatic planting of corn seeds which was released into the atmosphere by the exhaust. We believe that the major source of ditch water contamination is direct contamination by fugitive dust as drift during planting, re-distribution of surface residues during wind erosion, and preferential flow of soil surface residues through surface run-off. It would follow that mitigating fugitive dust during planting would have a significant impact on neonicotinoid residues reaching ditches and streams. Using the Bayer Fluency Agent [41] to mitigate fugitive dust, appeared to have no impact (Table 3) on the residues reaching ditch water. Effects may have been diluted by planting activities in neighbouring fields over which we had no control.

Planter modifications reduced fugitive clothianidin residues by 98% during planting [50]. Following our argument, a 90% reduction in dust through mitigation would lead to 90% reduction in ditch water contamination, diluting the maximum and mean concentration of clothianidin in ditch water from 7.2 and 1.11 ng/mL to 0.72 and 0.11 ng/mL. Diluting this again by 40-fold would result in concentrations in the order of 0.018 maximum and 0.003 mean ng/mL in stream water.

Mayflies are more sensitive to neonicotinoid insecticides than most other species [27, 28]. They were shown to have a chronic no-observed-effect-concentration (NOEC) of 0.3 ng/mL for thiamethoxam [27]. Assuming the toxicities of clothianidin and thiamethoxam to mayflies are broadly similar, these data support a 1.6 and 100-fold margin of safety to mayflies in most streams if fugitive dust on pneumatic planters were properly mitigated. In North America, insufficient action has been taken to mitigate fugitive dust from pneumatic planters to reduce contamination of surface water by neonicotinoid insecticides or any other pesticide applied to seeds.

## Conclusions

There is approximately a 40- to 50-fold dilution of neonicotinoid insecticide residues in surface waters found in streams compared with what is found in ditches draining the fields where seed treatments have been applied. While this study does not prove it, the data do suggest that most of the residues in ditch water appear to come from direct contamination by fugitive dust during planting, or indirectly as the settled dust is redistributed by wind or water erosion. We believe there is much less contamination of ditch water coming via leaching from planted, treated seed. We have shown that most of the escaping contaminated dust, also the primary source of contaminated water, can be eliminated by modifying pneumatic planters. We argue that these modifications to planters must be investigated, and doing so will greatly reduce the risk of exposure of aquatic non-targets to not only neonicotinoid insecticide seed treatments, but any pesticide applied to seed. Unfortunately there is currently little to no evidence that the industry or grain producers have adopted these modifications.

## Supporting information

S1 Table Limits of detection, quantification, percentage of recovery and optimized mass spectrometry parameters used for the LC-ESI(+)-MS/MS analysis of neonicotinoids in water with their relevant deuterium internal standard. (DOCX)

S2 Table Determination of 7 neonicotinoid insecticide and 2 herbicides residues in farm water (ng/mL) by QuEChERS method and LC-MS/MS SW Ontario, 2013. LOD and LOC reported at bottom. Values in black reported previously in PLOS NE|DOI:10.1371/ journal.pone.0118139. Values in red not previously reported.
(XLSX)
