# Supplementary material for: Neonicotinoid insecticide residues in subsurface drainage and open ditch water around maize fields in southwestern Ontario
Source: PLoS One. 2019 Apr 4;14(4):e0214787. doi: 10.1371/journal.pone.0214787 (PMC6449027; doi:10.1371/journal.pone.0214787)
Supplement: S1 Table — (DOCX) [file pone.0214787.s001.docx]

Supplementary Table S1. Limits of detection, quantification, percentage of recovery and optimized mass spectrometry parameters used for the LC-ESI(+)-MS/MS analysis of neonicotinoids in water with their relevant deuterium internal standard

| Analyte | R_t_^A^ | Precusor ion | Product ions^B^ | DP^C^ | FP^D^ | CE^E^ | CXP^F^ | LOD±SD^G^ | LOQ ±SD^H^ | %R±SD^I^ |
| --- | --- | --- | --- | --- | --- | --- | --- | --- | --- | --- |
|  | (min) | (*m/z*) | (*m/z*) | (V) | (V) | (V) | (V) | (ng/mL) | (ng/mL) | (%) |
| Clothianidin | 10.0 | 250.0 [M+H]^+^ | **131.8**/ 169.1 | 73.0 | 260.0 | 21.0/18.4 | 17.0/17.5 | 0.017±0.002 | 0.047±0.007 | 91.7±4.4 |
| Thiamethoxam | 8.0 | 292.3 [M+H]^+^ | **181.1**/ 211.1 | 43.0 | 142.0 | 31.5/19.0 | 22.0/18.5 | 0.004±0.001 | 0.011±0.002 | 92.6±3.1 |
| Imidacloprid | 9.4 | 256.0 [M+H]^+^ | **175.1**/ 209.0 | 39.0 | 143.0 | 21.0/18.0 | 16.0/20.0 | 0.011±0.001 | 0.031±0.004 | 78.9±0.6 |
| Thiacloprid | 11.4 | 252.9 [M+H]^+^ | **126.0**/ 186.0 | 40.0 | 165.5 | 32/0/19.2 | 14.0/24.0 | 0.002±0.0004 | 0.005±0.001 | 47.1±3.0 |
| Acetamiprid | 10.3 | 223.1 [M+H]^+^ | **126.0**/ 56.0 | 27.0 | 97.0 | 17.0/27.0 | 25.0/14.0 | 0.007±0.000 | 0.021±0.001 | 96.3±7.2 |
| Dinotefuran | 6.7 | 203.2 [M+H]^+^ | **129.0**/ 113.2 | 37.0 | 150.0 | 17.5/19.5 | 14.0/15.0 | 0.019±0.001 | 0.051±0.004 | 67.6±3.5 |
| Nitenpyram | 5.6 | 271.0 [M+H]^+^ | **99.0**/ 126.0 | 40.0 | 116.5 | 26.0/32.5 | 12.1/14.0 | 0.066±0.017 | 0.155±0.041 | 69.7±6.7 |
| Internal Standards | |  |  |  |  |  |  |  |  |  |
| Clothianidin-d3 | 10.0 | 253.0 [M+H]^+^ | **172.0**/ 131.8 | 73.0 | 260.0 | 21.0/18.4 | 21.0/18.4 |  |  |  |
| Thiamethoxan-d3 | 8.0 | 295.0 [M+H]^+^ | **184.1**/ 214.1 | 100.0 | 306.0 | 31.5/19.0 | 22.0/18.5 |  |  |  |
| Imidacloprid-d4 | 9.4 | 260.0 [M+H]^+^ | **179.1**/ 213.0 | 100.0 | 296.0 | 23.0/17.4 | 20.0/25.0 |  |  |  |
| Acetamiprid-d3 | 10.3 | 226.0 [M+H]^+^ | **126.0**/ 59.0 | 120.0 | 310.0 | 26.0/24.0 | 15.0/10.0 |  |  |  |

Retention time, ^B^**quantifier ion**/qualifier ion, ^C^declustering potential, ^D^focusing potential, ^E^collition energy, ^F^cell exit potential, ^G^limit of detection (S/N=3, n=10), ^H^limit of quantitation (S/N=10, n=10), ^I^percentage of recovery at 0.5 ng/mL (n=3)
